# Effect of Serum Heat-Inactivation and Dilution on Detection of Anti-WNV Antibodies in Mice by West Nile Virus E-protein Microsphere Immunoassay

**DOI:** 10.1371/journal.pone.0045851

**Published:** 2012-09-25

**Authors:** Madhuri Namekar, Mukesh Kumar, Maile O'Connell, Vivek R. Nerurkar

**Affiliations:** Department of Tropical Medicine, Medical Microbiology and Pharmacology, Pacific Center for Emerging Infectious Diseases Research, John A. Burns School of Medicine, University of Hawaii at Manoa, Honolulu, Hawaii, United States of America; Washington University, United States of America

## Abstract

Immunopathogenesis studies employing West Nile virus (WNV) mice model are important for the development of antivirals and vaccines against WNV. Since antibodies produced in mice early during WNV infection are essential for clearing virus from the periphery, it is important to detect early and persistent anti-WNV antibodies. ELISA and plaque reduction neutralization tests are traditionally used for detection of anti-WNV antibodies and WNV-neutralizing antibodies, respectively. Although these assays are sensitive and specific, they are expensive and time consuming. Microsphere immunoassays (MIA) are sensitive, specific, allow for high throughput, are cost effective, require less time to perform than other methods, and require low serum volumes. Several assay parameters such as serum heat-inactivation (HI) and dilution can alter WNV MIA sensitivity. We examined the effect of these parameters on WNV E-protein MIA (WNV E-MIA) for the enhanced detection of anti-WNV IgM and IgG antibodies. WNV E-MIA was conducted using serial dilutions of HI and non-HI (NHI) serum collected at various time points from mice inoculated with WNV. HI significantly enhanced detection of IgM and IgG antibodies as compared to NHI serum. WNV IgM and IgG antibodies in HI sera were detected earlier at day 3 and IgM antibodies persisted up to day 24 after infection. HI serum at 1∶20 dilution was found to be optimal for detection of both IgM and IgG antibodies as compared to higher-serum dilutions. Further, addition of exogenous complement to the HI serum decreased the WNV E-MIA sensitivity. These results suggest that serum-HI and optimal dilution enhance WNV E-MIA sensitivity by eliminating the complement interference, thereby detecting low-titer anti-WNV antibodies during early and late phases of infection. This improved MIA can also be readily employed for detection of low-titer antibodies for detection of other infectious agents and host proteins.

## Introduction

West Nile virus (WNV), a mosquito-borne flavivirus that causes lethal encephalitis, has emerged as a significant cause of viral encephalitis in the United States [1]. Although, WNV infection in humans is mainly acquired after mosquito bite, human-to-human transmission can occur through blood transfusion, organ transplantation and breastfeeding [2], [3]. Currently, no antiviral or vaccine is available to counteract or protect against WNV infection in humans [4]. WNV immunopathogenesis studies in animal models such as mice provide important information for the development of antivirals and vaccines against WNV infection in humans. In WNV-infected mice, IgM and IgG antibodies are produced early after the infection and persist for a long time. These WNV-specific antibodies limit viremia and dissemination of virus into the CNS and provide protection against lethal infection [5]. Induction of these antibodies is also a critical determinant for the efficacy of WNV vaccines [4]. Therefore, it is important to detect low levels of both anti-WNV IgM and IgG antibodies during early and late phase of the infection. WNV E-protein enzyme linked immunosorbent assay (ELISA) and plaque reduction neutralization test (PRNT) have been used for detection of both anti-WNV IgM and IgG antibodies, and WNV-neutralizing antibodies, respectively, in mice [6]. Though these assays are sensitive and specific, they are expensive and time consuming. Luminex-based microsphere immunoassays (MIA) have been developed and used for detection of anti-WNV antibodies in humans and in mice model using purified recombinant proteins (E, NS3 and NS5) of WNV [7]–[11]. WNV E-MIA is sensitive, cost-effective and requires less time than traditional ELISA and PRNT assays for detection of anti-WNV antibodies [7], [8]. MIA has also been used for improved serological detection of several other viruses such as respiratory syncytial virus [12], HIV [13], WNV [7], [8], [14], human papillomaviruses [15], equine arteritis virus [16], and avian influenza virus [17].

Several assay parameters such as heat-inactivation (HI) of serum and serum dilution can affect the MIA results. Heat-inactivation of serum at 56°C for 30 min is a standard procedure in diagnostic laboratories to conduct neutralization test for the purpose of inactivation of complement [18]. Complement components present in serum are known to react with multi-molecular immune complexes or immunoglobulin aggregates [19], [20]. Serum heat-inactivation decreased the number of false-positives in multiplexed immunoassay for detection of antibodies against human papilloma viruses [15]. In another Luminex based assay for detection of human leukocyte antigen (HLA) antibodies, HI serum decreased the frequency of false-negative results by eliminating the complement interference or prozone effect [21]. In contrast, HI of the cattle serum had little effect on the performance of the liquid array multiplexed assay for detection of antibodies against foot and mouth disease virus [22]. Two recent WNV persistence studies have employed WNV E-MIA to study the anti-WNV antibody response in the mice after infection [10], [11]. In the first study, MIA was conducted using non heat-inactivated (NHI) sera [10], whereas in the second study, sera were HI at 56°C for 1 hour prior to testing [11]. These two studies detected total anti-WNV antibodies (IgG, IgA and IgM). Therefore, the effect of HI sera on the WNV E-MIA for detection of low-titer anti-WNV IgM and IgG during early and late phase of infection cannot be deduced. In addition to HI, serum dilution is another important parameter that can affect the sensitivity of MIA. Serum dilution, to some extent can eliminate the complement interference or prozone effect observed in the case of high antibody titers, thus can improve the sensitivity of MIA [21]. On the other hand increased serum dilution can lead to decreased sensitivity [23]. WNV E-MIA was conducted at 1∶100 dilution of serum [8], [10], [11], whereas other MIA employed lower serum dilution (1∶20), as it resulted in the low levels of non-specific background with high dynamic range of signal intensities [24], [25]. Therefore, HI of serum and serum dilution are the critical parameters that can affect MIA results and thus should be considered as important part of the development and optimization of MIA.

In this study, we examined the effect of serum- HI and -dilution on the sensitivity of WNV E-MIA for the detection of anti-WNV IgM and IgG antibodies in mice.

## Materials and Methods

### Ethics Statement

This study was specifically approved by the University of Hawaii Institutional Animal Care and Use Committee (protocol number 10-948) and conducted in strict accordance with animal use protocols in the animal biosafety level-3 laboratory. Mice that exhibited severe disease were euthanized to limit suffering.

### WNV Infection

Nine-week old C57BL/6 mice were purchased from the Jackson Laboratory (Bar Harbour, ME). All infection experiments were conducted using the lineage I WNV strain (NY 99) as described previously [26]. Lineage I WNV strain NY99 used in all experiments was originally isolated from a crow in New York and further propagated in Vero cells as described previously [27]. Mice were inoculated via the footpad route with 100 PFU of WNV or with PBS (mock). On days 0, 3, 6, 8, 10 and 24 after infection, 100 µL blood was collected from tail vein, from which serum was separated and frozen at −80°C for future analyses.

### Coupling of Microspheres with rWNV-E Antigen

Magnetic carboxylated microspheres (MagPlexTM-C) were obtained from Luminex Corporation (Austin, TX, USA). A two-step carbodiimide process recommended by Luminex Corporation (Austin, TX) was used to link 10 µg of purified rWNV-E (L2 Diagnostics) to the surface of 1.25×10^6^ microspheres as described previously [8]. *Drosophila* S2 expression system was employed by L2 Diagnostics to make the rWNV-E antigen [28]. The antigen-conjugated microspheres were stored in 250 µL of PBN buffer (PBS with 1% bovine serum albumin Fraction V, OmniPur, and 0.05% Ultra sodium azide, Sigma Aldrich) at 4°C.

### WNV E-MIA

Serum samples were diluted in PBS-1% BSA. A total of 50 µL of PBS-1% BSA containing approximately 1,250 coupled microspheres were added to each well of a flat-bottom 96-well plate. Fifty µL of diluted serum was added to the beads and incubated for 30 min in the dark. The plates were then washed twice with 200 µL of PBS-1% BSA and 50 µL of diluted red-phycoerythrin (R-PE) conjugated secondary antibody (2 µg/mL) was added to test wells and incubated for 45 min in the dark. The plates were washed twice with 200 µL of PBS-1% BSA. Microspheres were then resuspended in 100 µL of PBS-1% BSA per well and incubated for 5 min before analysis on the Luminex 100 machine (Qiagen, Valencia, CA). The median fluorescence intensity (MFI) was quantitated for 100 microspheres and recorded for each well. The secondary antibodies used were R-PE conjugated F(ab’)2 fragment goat anti-mouse IgG, Fcγ fragment specific and R-PE conjugated F(ab’)2 fragment goat anti-mouse IgM, µ chain specific (Jackson Immunoresearch, West Grove, PA). All assays were done in duplicate and all incubations were conducted on a plate shaker at 700 rpm and the wash steps were conducted using 96-well magnetic plate separator (Millipore Corp., Billerica, MA).

To study the effect of HI on the sensitivity of WNV E-MIA, serum samples were HI at 56°C for 30 min in water bath. To optimize serum dilution, WNV E-MIA was conducted using HI and NHI mice sera serially diluted from 1∶20 to 1∶160 using PBS-1% BSA.

### Cutoff Determinations

Cutoff values were calculated as the average MFI of 25 serum samples from mock-infected C57BL/6 mice plus three standard deviations (Microsoft® Office Excel). Negative cutoff values for HI and NHI serum for IgG MFI were 131 and 85, and for IgM MFI were 366 and 314, respectively. Serum samples with MFI values greater than the cutoff were considered positive. Control beads were coupled with 1X PBS instead of WNV E-protein using the aforementioned coupling protocol to check for nonspecific attachment of serum proteins to the microspheres. Control beads mean IgG MFI for HI and NHI serum samples at 1∶20 dilution were 18 and 11, and the IgM MFI were 27 and 63, respectively, which suggests that the nonspecific attachment of the serum proteins to the microspheres was minimal.

### Complement Addition to HI Serum

HI serum was diluted 1∶20 in PBS-1% BSA and 4 U of reconstituted guinea pig complement (C’) (Sigma-Aldrich) was added to 240 µL of diluted serum. In a separate group, HI serum after addition of C’ were again heat-inactivated at 56°C for 30 min to inactivate the complement. HI serum without C’, HI serum with C’ and re-heat-inactivated HI serum with C’ were tested by WNV E-MIA for detection of anti-WNV IgM and IgG antibodies.

### Plaque Reduction Neutralization Test (PRNT)

Serum samples used for conducting MIA were also used to screen for anti-WNV neutralizing antibodies by PRNT. Both NHI and HI sera were diluted serially from 1∶20 to 1∶160 and PRNT was conducted by using lineage I WNV strain (NY99), as described previously [27], [29].

### Statistical Analysis

All data are reported as mean MFI ± standard deviation of at least two independent experiments conducted in duplicate using GraphPad Prism 5.0 software.

## Results and Discussion

### HI of Serum Enhanced the Detection of Anti-WNV IgM and IgG Antibodies in Mice

**Figure 1:**
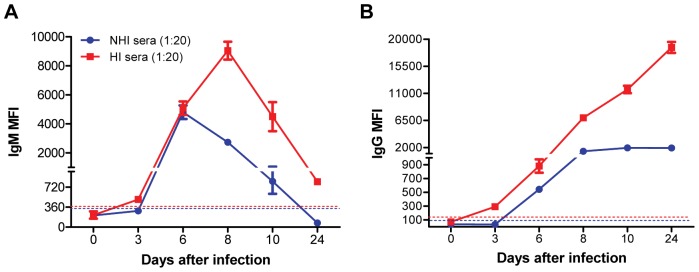
Effect of serum heat-inactivation on the detection of anti-WNV IgM and IgG antibodies in mice. Adult C57BL/6 mice were inoculated subcutaneously with 100 PFU of WNV. Mice (n = 10 per time point) were bled at 0, 3, 6, 8, 10 and 24 days after infection and serum was separated, and same time point serum was pooled. Serum samples were HI at 56°C for 30 min. NHI and HI sera were diluted 1∶20 in PBS-1% BSA and were tested by WNV E-MIA for the presence of anti-WNV (**A**) IgM and (**B**) IgG antibodies. Results are reported as MFI per 100 microspheres. Data are expressed as MFI ± SD and is representative of three independent experiments conducted in duplicate. Dotted line indicates the cutoff value. HI serum is depicted by red line and NHI serum by blue line.

First, we examined the effect of HI of serum on the WNV E-MIA using both NHI serum and HI serum at 1∶20 dilution. Using HI serum anti-WNV IgM first appeared at day 3 peaked at day 8 and then gradually decreased at days 10 and 24 after infection (Figure 1A). Similarly, anti-WNV IgG first appeared at day 3 and then demonstrated gradual increase up to day 24 after infection (Figure 1B). HI of serum enhanced the detection of anti-WNV IgM and IgG antibodies as there was approximately 2 to 10 fold increase in IgM and IgG MFI in HI serum as compared to NHI serum. WNV E-MIA optimized in this study is much more precise than traditional ELISA methods used for detection of anti-WNV antibodies in mice wherein anti-WNV IgM was first detected at day 4 after infection and anti-WNV IgG was first detected at day 6 after infection [6]. These WNV-specific ELISA methods were also conducted using HI serum [30]. In addition, two WNV studies using animal models [11], [31] demonstrated persistence of IgM antibody secreting cells or IgM antibodies until day 14 and 18 after infection in mice [11] and hamster [31], respectively. Similarly, persistence of WNV IgM antibodies was demonstrated in patients with WNV encephalitis for more than a year [32], [33]. Our results demonstrate that IgM antibodies persist in WNV-infected mice until day 24 after infection, albeit at very low level suggesting that HI of serum enhances WNV E-MIA for the detection of anti-WNV IgM and IgG antibodies in mice. This increase in the MFI may be primarily due to the elimination of complement interference present in the serum as a result of heat treatment at 56°C for 30 min. However, heat treatment of samples may also change the accessibility of the antibody epitopes in a complex serum sample, thus can improve the performance of MIA [34]. In addition, previous studies using mouse models have employed polyclonal secondary antibody, which detect total anti-WNV antibodies (IgM, IgG, IgA) [10], [11]. Whereas, in this study we employed monoclonal antibodies to independently detect anti-WNV IgM and IgG antibodies.

MIA data can be represented using various methods [7], [8], [10], [11], [35]. Signal-to-noise ratios is a common method of presenting data, where negative control sera are used as the denominator [8], or where test sample reacted on a control antigen is used as a denominator [35]. Often higher dilutions than those reported by us are advantageous and may give better results for detection of WNV antibodies in human [8], [14], [35]. However, using this method false-negatives could potentially exist because non-specific reactions with the negative control antigen could generate artificially low numbers in the data transformation scheme [35]. Since detection of WNV antibodies using MIA in mice used MFI for data representation [10], [11], we used this method to represent our data for comparison with existing data on detection of anti-WNV antibodies in mice.

To confirm the presence of anti-WNV neutralizing antibodies in serum from WNV-infected mice after heat-inactivation and dilution, we conducted PRNT on the same serum samples used for MIA using both NHI and HI sera diluted serially from 1∶20 to 1∶160. As expected, high levels of anti-WNV neutralizing antibodies were detected at days 6, 8, 10 and 24 after infection. PRNT, even though it is specific, did not detect very low levels of antibodies produced at day 3 after infection (Figure S1). However, WNV E-MIA detected these very low levels of antibodies produced early during infection (Figure 1).

### Serum Dilution Enhances Detection of Anti-WNV IgG, but not IgM Antibody

**Figure 2:**
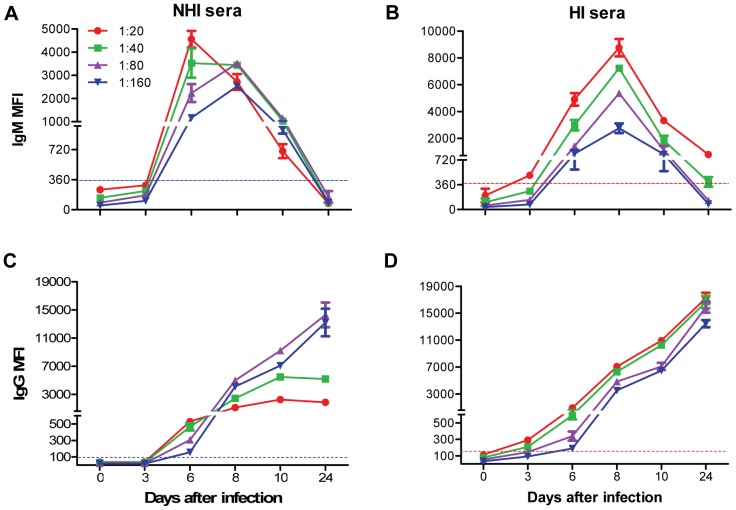
Effect of serum dilution on the detection of anti-WNV IgM and IgG antibodies in mice. NHI and HI sera at indicated time-points after infection were diluted serially from 1∶20 to 1∶160 in PBS-1% BSA and were tested by WNV E-MIA for the presence of anti-WNV (**A and B**) IgM and (**C and D**) IgG antibodies. Data are expressed as MFI ± SD and is representative of two independent experiments conducted in duplicate. Dotted line indicates the cutoff value. HI serum is depicted by red line and NHI serum by blue line.

Another important parameter that can affect the performance of Luminex-based serological assays is the dilution of the serum. Serum dilution, though it contributes to low background signal, can also lead to decrease in sensitivity of the Luminex assay [23] and false-negatives could potentially exist [35]. In the previous studies, WNV E-MIA was conducted at a serum dilution of 1∶100 as it provided low background binding and optimal assay results [8], [10], [11], [36]. However, these higher serum dilutions might have also decreased the sensitivity as well. Therefore, to determine the optimum serum dilution for WNV E-MIA, NHI and HI sera were serially diluted from 1∶20 to 1∶160 and anti-WNV IgM and IgG antibodies were detected. IgM MFI decreased with increase in serum dilution of NHI serum at day 6 after infection. However, there was very little increase in IgM MFI for higher dilutions (1∶40 and 1∶80) of NHI serum at days 8 and 10 after infection. No IgM was detected at days 3 and 24 after infection at any dilution of NHI serum (Figure 2A). In contrast, IgM MFI of HI serum at various time-points after infection was higher at 1∶20 dilution, which gradually decreased with increase in serum dilution. HI serum at 1∶20 dilution demonstrated improved sensitivity for IgM detection, particularly at days 3 and 24 after infection, where the IgM antibody levels are usually very low (Figure 2B). IgG MFI for NHI serum increased with increase in serum dilution at days 8, 10 and 24, while it was decreased at day 6 after infection (Figure 2C). Similar to IgM, IgG MFI for HI serum was highest for 1∶20 dilution and then gradually decreased at 1∶40, 1∶80 and 1∶160 dilutions (Figure 2D). Interestingly, IgG MFI obtained for higher dilutions (1∶80 and 1∶160) of the NHI serum at days 8, 10 and 24 are almost similar to that obtained for 1∶20 and 1∶40 dilutions of HI serum. Thus dilution of NHI serum enhanced the detection of anti-WNV IgG antibodies, however, the same effect was not observed for IgM antibodies. This observed change in MFI may be due to the prozone effect or high-dose hook effect as a result of high antibody titers in serum or the complement interference in the NHI serum. Prozone effect and complement interference in the immunoassays can be eliminated by sufficient dilution of the serum [23], [37], but this can also lead to decrease in sensitivity of the assay, as observed for IgM antibodies (Figure 2A). Therefore, we determined 1∶20 to be the optimal dilution of HI serum for WNV E-MIA that can detect low-titer IgM and IgG antibodies during early and late time-points after infection.

**Figure 3:**
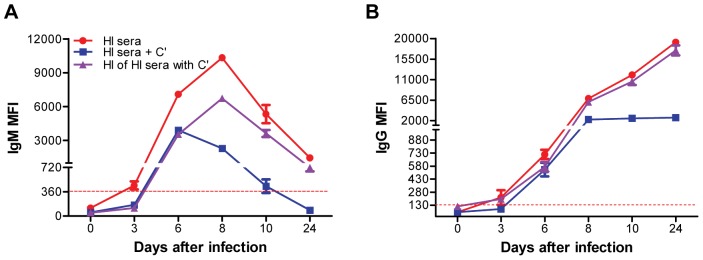
Effect of exogenous addition of complement to heat-inactivated mice serum in WNV E-MIA. HI serum at indicated time-points after infection was diluted 1∶20 in PBS-1% BSA and 4 U of reconstituted guinea pig complement (C’) was added to 240 µL-diluted serum. Also, HI serum after addition of C’ was again heat-inactivated at 56°C for 30 min to inactivate the complement. HI serum without C’, with C’ and heat inactivation of HI serum with C’ were tested by WNV E-MIA for detection of anti-WNV (**A**) IgM and (**B**) IgG antibodies. Data are expressed as MFI ± SD and is representative of two independent experiments conducted in duplicate. Dotted line indicates the cutoff value. HI serum is depicted by red line.

### Complement Interferes in WNV E-MIA

To validate the role of complement interference in WNV E-MIA, we added exogenous guinea pig complement to the 1∶20 dilution of the HI serum and then conducted WNV E-MIA. Addition of complement resulted in decrease in both IgM and IgG MFI at days 3, 6, 8, 10 and 24 after infection (Figure 3A and 3B). Surprisingly, IgM and IgG MFI obtained after complement addition to HI serum were almost similar to those obtained for NHI serum from WNV-infected mice (Figure 1). Further, to test if re-heat inactivation of HI serum with complement will restore the MFI values we re-heat-inactivated HI serum with complement and conducted WNV E-MIA. Interestingly, IgM MFI after heat-inactivation of HI serum with complement at days 8, 10 and 24 was increased as compared to non heat-inactivated HI serum with complement, but it did not restore completely, and remained unchanged for days 3 and 6 after infection (Figure 3A). Similarly, IgG MFI after heat-inactivation of HI serum with complement was increased at days 8, 10 and 24 after infection as compared to non heat-inactivated HI serum with complement (Figure 3B). However, in contrast to IgM, IgG MFI was restored after heat-inactivation of HI serum with complement. The possible reason for this difference may be due to aggregation of IgM antibodies as a result of prolonged heat treatment at 56° for 1 hr. Mouse IgM antibodies are more sensitive to heat treatment whereas IgG1 and IgG2a antibodies are relatively heat-resistant [38]. Collectively, these results suggest that complement interferes in WNV E-MIA, thus causing the false-negative or false-low positive results. In addition, these results confirm the role of complement in causing the prozone effect in high antibody titer serum samples as fold increase in IgM and IgG MFI was higher for HI serum at days 8, 10 and 24 compared to the NHI serum (Figure 1A and 1B). Similarly, Schnaidt et al [21] demonstrated that complement component C1 can cause prozone effect in the case of high antibody titers in Luminex based assay for HLA antibody detection. Complement can interfere in Luminex assays by binding to two or more closely spaced antibodies immobilized on a solid surface, which is only possible in the case of high antibody titers, but not with low antibody titer serum samples [21]. This complement binding blocks the Fc portion of antigen-specific antibodies and competitively prevents the binding of secondary antibodies, eventually giving false-negative results [21], [23], [39].

### Conclusions

This study for the first time demonstrates that HI of the serum contributes to enhanced detection of anti-WNV IgM and IgG antibodies using MIA. The WNV E-MIA optimized in our laboratory is robust, sensitive and high throughput assay for detection of anti-WNV IgM and IgG antibodies in mice, particularly at early and late time-points after WNV infection in mice. These findings led us to include a HI step of 56°C for 30 min and 1∶20 as optimum serum dilution, as part of our sample preparation procedures for conducting WNV E-MIA in mice. These data strongly suggest that HI of serum and optimized serum dilution should be considered as one of the important parameter during development and optimization of other Luminex-based MIA.

## Supporting Information

Figure S1 **Plaque reduction neutralization test (PRNT) for detection of anti-WNV neutralizing antibodies in mice.** NHI and HI sera from WNV-infected mice collected at 0, 3, 6, 8, 10 and 24 days after infection were serially diluted from 1∶20 to 1∶160 and PRNT was conducted. Percent reduction in number of plaques obtained per time point was calculated for both (**A**) NHI and (**B**) HI sera. Data are expressed as average percent reduction in number of plaques per time point conducted in duplicate.
(TIF)
