# Supplementary figures and images for: Effect of Serum Heat-Inactivation and Dilution on Detection of Anti-WNV Antibodies in Mice by West Nile Virus E-protein Microsphere Immunoassay
Source: PLoS One. 2012 Sep 25;7(9):e45851. doi: 10.1371/journal.pone.0045851 (PMC3457982; doi:10.1371/journal.pone.0045851)

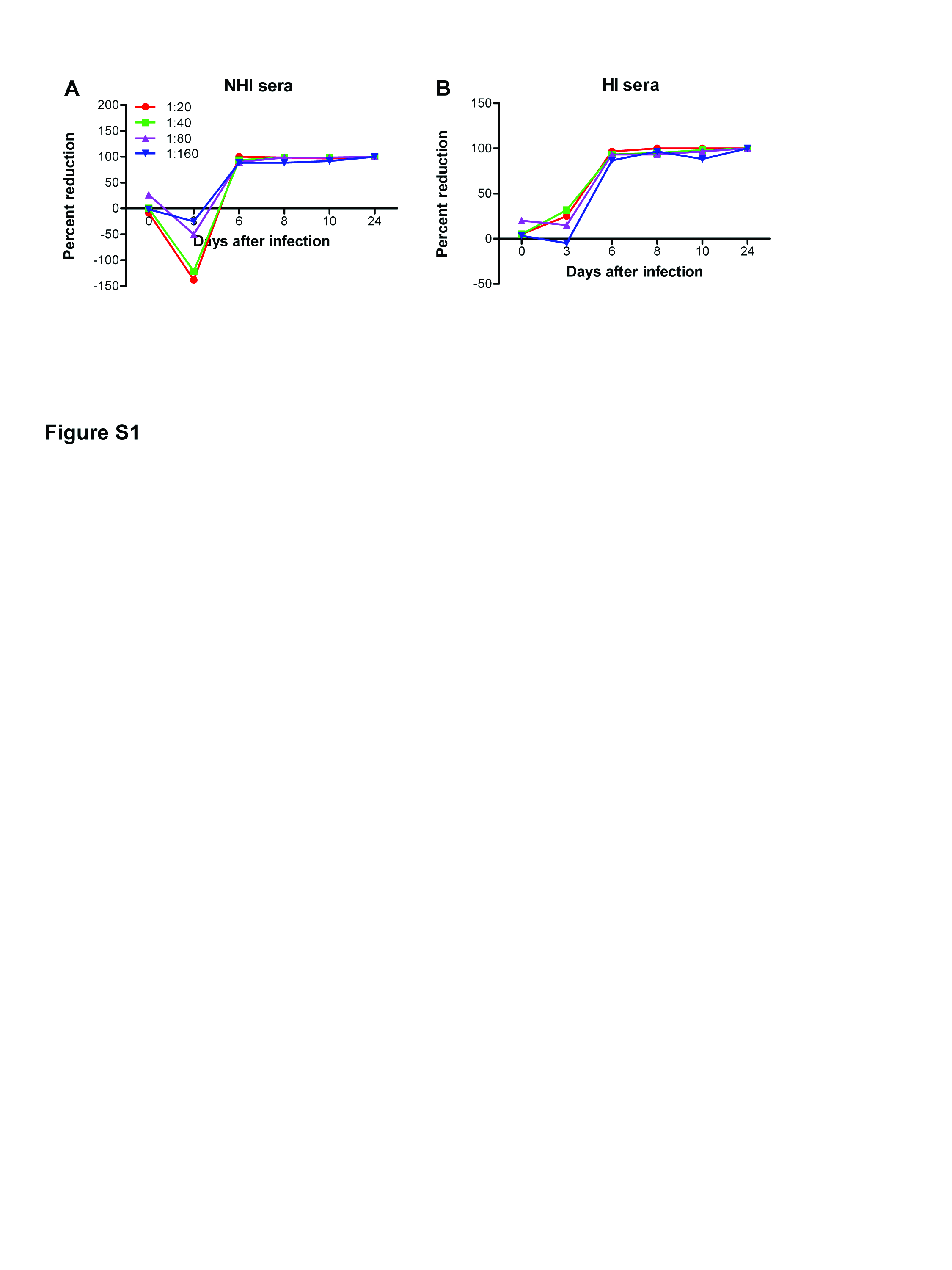

Supplement: Figure S1 — Plaque reduction neutralization test (PRNT) for detection of anti-WNV neutralizing antibodies in mice. NHI and HI sera from WNV-infected mice collected at 0, 3, 6, 8, 10 and 24 days after infection were serially diluted from 1∶20 to 1∶160 and PRNT was conducted. Percent reduction in number of plaques obtained per time point was calculated for both (A) NHI and (B) HI sera. Data are expressed as average percent reduction in number of plaques per time point conducted in duplicate. (TIF) [file pone.0045851.s001.tif]
